# Penicillin in Diseases of the Ear, Nose, and Throat

**Published:** 1947

**Authors:** E. Watson-Williams

**Affiliations:** Surgeon in charge, Ear, Nose and Throat Department, Bristol Royal Hospital. Lecturer in Diseases of Ear, Nose and Throat, University of Bristol


### PENICILLIN IN DISEASES OF THE EAR, NOSE, AND THROAT

An introduction to a discussion by the Society on Wednesday, March 12th, 1947.

BY

E. Watson-Williams, M.C., Ch.M.,
*Surgeon in charge, Ear, Nose and Throat Department, Bristol Royal Hospital.*
*Lecturer in Diseases of Ear, Nose and Throat, University of Bristol.*

---

There is no question that, in such serious conditions as septicaemia or suppurative meningitis, the value of penicillin is established. In this paper, however, I shall not attempt to cover the whole subject of the uses of penicillin: rather I hope to indicate the use of this preparation in the common diseases of the ear, nose and throat.

### Ear

*Otitis externa.* Penicillin drops applied to the external meatus appear to me to have no value at all. In otitis externa it must be almost impossible by such means to maintain a sufficient concentration of the drug in contact with the infected tissues. At all events, among some thirty cases I have seen none in which it has given any good result: and in several I had the impression that the applications were actually irritating.

*Otitis media, acute.* Still less can penicillin drops be expected to achieve anything in otorrhea due to otitis media. Consider for a moment the anatomical features. In the normal ear there is an open passage, the aditus, between tympanum and mastoid antrum; in otitis media, by the time there is sufficient pus in the former to bulge the membrane it is inevitable that the infection has *already* reached the antrum—all clinical experience supports this expectation. Once this has occurred, or at least before perforation takes place, the mucosa of the aditus, antrum and cells swells up, obliterating the air spaces. How can it be possible that *any* bactericidal agent placed in the meatus can penetrate the perforation, often minute, pass up between the folds of swollen mucosa to the attic, and back through the aditus to the antrum and cells? Still less, how can it possibly reach the bacteria in the mucosa of these cells, and in effective concentration?

On the other hand the bacteria responsible for otitis media are almost without exception penicillin-sensitive. Penicillin given by intra-muscular injection will abort otitis media in a manner simply miraculous. I usually give 20,000 units three-hourly for the first twenty-four hours and half this dose for the next four to eight days ; it is seldom required after the fifth day. I have used it now in over one hundred and seventy cases, with hardly a failure. Indeed, my house surgeon recently complained that during five months he had only seen three cases that needed opening of the mastoid. Even where there is extensive post-aural oedema, or a periosteal abcess, an incision is all that is usually needed if penicillin is given. One must, of course, be guided as to the need for operation exactly as if no penicillin is being used. If either pain *or* tenderness *or* fever are not improving forty-eight hours after treatment has been started it is probable that an actual mastoid abscess exists which the drug is not reaching, and operation may be required.

Miss Z . . . a lady of forty-eight, matron of a county hospital, had a left acute otitis media after influenza ; she had never previously had any ear disease. Myringotomy was performed and full doses of sulphathiazole were given. After two days, pain and fever were still present, so penicillin was started. On the fifth day the pain and fever which had improved a little were again severe. I was asked to see her, as her own surgeon had urged her to allow the mastoid to be opened and she wanted to avoid an operation. I could only concur with his advice : the operation was performed and pus under pressure was found in the antrum—there was no extension of the disease (no " complication ") : recovery uninterrupted. The amount of pus was an unusual feature in the case just mentioned : my experience of cases treated with penicillin is that if an operation becomes necessary there is usually little or no pus—rather a puzzling feature until one becomes used to it.

### NOSE

*Local Application.* I have not found penicillin ointment so useful as sulphonamide preparations : it is true that I have treated only a few cases with penicillin. I have not used penicillin as a nasal spray : it is a method of some value, since after spraying the nose with ten per cent. penicillin one can demonstrate penicillin in the serum. But if a systemic effect is required intra-muscular injection is the only method worth considering : for severe sinusitis this is of great value. I shall be interested to hear what results others have obtained by inhalation methods in lung abscess, etc.

*Acute sinusitis.* The great majority of cases of acute sinusitis resolve without the need for operation and I cannot say whether the drug materially influences the course of events. Certainly patients for whom it is used seem to get rid of pain and fever more quickly than used to be the case : but most of these cases occur as complications of influenza, a disease which varies very much from time to time in the severity of its complications—which makes it difficult to reach a conclusion as to the value of any particular mode of treatment except after considerable experience.

*Chronic sinusitis.* I have used penicillin on a number of occasions when washing out chronically diseased sinuses. The three preparations used were a cream ; a jelly—I believe made with tragacanth ; and a glycerine-and-bismuth-subnitrate paste. I cannot say that I have found the addition of penicillin has had any effect at all. In three out of forty-one cases the symptons were improved for a few weeks but did not respond a second time. In some of the others a short improvement was noticed, but this is not uncommon whatever preparation is used. It is obviously a matter of great difficulty to get any effective concentration of penicillin into the sinuses in such a form that the drug will remain where it is placed for more than a very few hours.

### THROAT

Pencillin lozenges are becoming quite popular : almost everyone with a sore throat expects them. In simple acute streptococcal tonsillitis such lozenges appear to have definite value. The technical difficulties have been considerable ; the lozenge base must be sufficiently soluble to set free the penicillin fast enough to reach an effective concentration in the saliva, yet not so soft that the lozenge dissolves in a few minutes. One lozenge an hour is required and it should take an hour or nearly an hour to dissolve.

And here we meet another trouble, stomatitis. I have seen quite a number of cases now, with painful, pink, sore tongue and lips. Some maintain the effect is due to the composition of the lozenge base ; others that penicillin alters the natural flora of the mouth and so exposes the mucous membrane to unbalanced action of penicillin-resistant organisms. Against this, one does not get this trouble when giving penicillin by injection. I have been led to believe that penicillin itself is irritating to the mucous membrane. The lozenges should not be used for more than three days. My personal feeling is that it is a mistake to use penicillin in this indiscriminate fashion : most of the cases will respond just as rapidly to old-established methods of treatment. Where penicillin lozenges appear to have definite value is in a case of Vincent's Angina, which does not rapidly respond to Mist. Arsenicalis. I have had five such cases, in each of which the throat infection responded rapidly to lozenges. On the other hand, it is useless to rely on the lozenges alone. I have seen four patients whose ulceration progressed unhindered while they were using lozenges. Three cleared up at once on arsenic : the fourth was a syphilitic who had been treated with penicillin injections, followed by bismuth ; he had obvious bismuth stomatitis—blue line on gums and round a sloughy ulcer in the right tonsil. The ulceration was steadily progressing while he used penicillin lozenges : I referred him to the surgeon who had been giving the bismuth and have not heard of subsequent progress.

